# Quantitative Trait Loci for Seed Size Variation in Cucurbits – A Review

**DOI:** 10.3389/fpls.2020.00304

**Published:** 2020-03-20

**Authors:** Yu Guo, Meiling Gao, Xiaoxue Liang, Ming Xu, Xiaosong Liu, Yanling Zhang, Xiujie Liu, Jixiu Liu, Yue Gao, Shuping Qu, Feishi Luan

**Affiliations:** ^1^College of Life Sciences, Agriculture and Forestry, Qiqihar University, Qiqihar, China; ^2^Heilongjiang Provincial Key Laboratory of Resistance Gene Engineering and Preservation of Biodiversity in Cold Areas, Qiqihar, China; ^3^Qiqihar Horticultural Research Institute, Qiqihar, China; ^4^College of Horticulture, Landscape Architecture, Northeast Agricultural University, Harbin, China

**Keywords:** cucurbits, watermelon, pumpkin/squash, cucumber, melon, seed size, QTL, comparative analysis

## Abstract

Cucurbits (Cucurbitaceae family) include many economically important fruit vegetable crops such as watermelon, pumpkin/squash, cucumber, and melon. Seed size (SS) is an important trait in cucurbits breeding, which is controlled by quantitative trait loci (QTL). Recent advances have deciphered several signaling pathways underlying seed size variation in model plants such as Arabidopsis and rice, but little is known on the genetic basis of SS variation in cucurbits. Here we conducted literature review on seed size QTL identified in watermelon, pumpkin/squash, cucumber and melon, and inferred 14, 9 and 13 consensus SS QTL based on their physical positions in respective draft genomes. Among them, four from watermelon (*ClSS2.2*, *ClSS6.1*, *ClSS6.2*, and *ClSS8.2*), two from cucumber (*CsSS4.1* and *CsSS5.1*), and one from melon (*CmSS11.1*) were major-effect, stable QTL for seed size and weight. Whole genome sequence alignment revealed that these major-effect QTL were located in syntenic regions across different genomes suggesting possible structural and functional conservation of some important genes for seed size control in cucurbit crops. Annotation of genes in the four watermelon consensus SS QTL regions identified genes that are known to play important roles in seed size control including members of the zinc finger protein and the E3 ubiquitin-protein ligase families. The present work highlights the utility of comparative analysis in understanding the genetic basis of seed size variation, which may help future mapping and cloning of seed size QTL in cucurbits.

## Introduction

Seed is the start and end points of plant life, and the important determinants of growth and development. Seed size is a key agronomic characteristic of evolutionary fitness in plants during domestication and breeding in many crops with seeds as the main product organ, and a key factor affecting seed yield, eating quality, tolerances to environmental stresses (Song et al., 2007; Bai et al., 2010; Gu et al., 2017; Smitchger and Weeden, 2018). In general, large seeds have more advantages than small ones in crop production because large seeds have bigger endosperm or cotyledons that can provide more nutrients for seedling establishment (Chen et al., 2006). Large seeds also have faster germination rate than small seeds, resulting in stronger seedlings that can better compete for light and nutrition, and stronger resistance or tolerance to adverse environmental conditions (Silvertown, 1981; Coomes and Grubb, 2003; Gómez, 2004). On the other hand, small seeds may have an advantage in seed transmission, thus affect the reproductive capacity of offspring (Wu et al., 2006).

For many crops, seed size is an important target of selection during domestication (Xia et al., 2018). The seeds of wild plants of modern crops are usually small and roundish in shape, while the domesticated ones in general are much larger. Changes in seed size are the results of natural selection and artificial selection in adaptation to different environments or human needs during domestication. Seed size varies greatly among crop plants (Stanton, 1984; Gómez, 2004; Moles et al., 2005). For crops with seeds as the final target of production (for example, rice, wheat and soybean), seed size is also the determinant of productivity and yield (Fan et al., 2006; Chettoor et al., 2016; Hu et al., 2018). As such, increase of seed size has been one of the primary goals in breeding in cereal crops (Zhang et al., 2016; Savadi, 2018).

**FIGURE 1:**
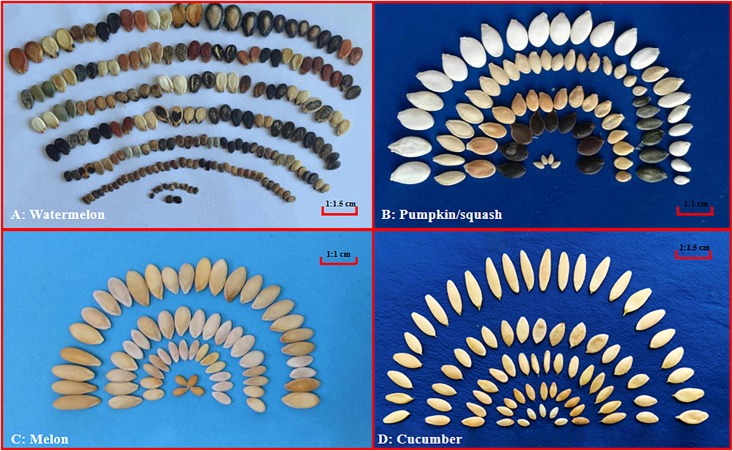
Seed size variation among cucurbit crops. Representative seeds of watermelon (*Citrullus lanatus*), pumpkin/squash (*Cucurbita*), melon (*Cucumis melo*) and cucumber (*Cucumis sativus*) are shown in **(A–D)**, respectively. All images were taken by the authors.

Crops in the Cucurbitaceae family, which often referred to as ‘cucurbits,’ include several economically important fruit vegetables worldwide such as watermelon [*Citrullus lanatus* (Thunb.) Matsum. & Nakai], melon (*Cucumis melo* L.), cucumber (*Cucumis sativus* L.), and pumpkin/squash (*Cucurbita* spp.). Cucurbits are generally prized for their delicious fruits, and the seeds are also good sources of vegetable oil and proteins. For example, the watermelon seeds are rich in oil (Baboli and Kordi, 2010) and protein (Al-Khalifa, 1996; Baboli and Kordi, 2010). However, seed size and weight within cultivated cucurbits also vary considerably depending on their intended uses, which is especially true in watermelon and pumpkin whose seeds have dual uses and seed size is an important target in breeding (Silvertown, 1981; Xiang et al., 2002; Tian et al., 2012). On the other hand, seeds of melon and cucumber do not seem to be under selection in modern breeding, thus which do not seem show as much diversity in seed size and color as those in watermelon and pumpkin/squash (Figure 1). Therefore, the cucurbits may offer a good system to understand the differential roles of artificial selection for seed-related traits during cucurbits breeding.

In cucurbits, seed size and weight are quantitatively inherited in nature, and the genetic basis is still poorly understood. However, in recent years, rapid progress has been made in genome sequencing among cucurbits. So far draft genomes have been developed for cucumber (Huang et al., 2009; Yang L. M. et al., 2012; Li Q. et al., 2019), melon (Garcia-Mas et al., 2012; Ruggieri et al., 2018), watermelon (Guo et al., 2013), bottle gourd, *Lagenaria siceraria* (Wu et al., 2017), bitter gourd, *Momordica charantia* (Urasaki et al., 2017), pumpkin/squash (Sun et al., 2017; Montero-Pau et al., 2018), and wax gourd, *Benincasa hispida* (Thunb.) Cogn. (Xie et al., 2019). These advances have made it possible to understand the genetic architecture of seed size variation in cucurbits through QTL studies and genome-wide association analysis. Thus in this report, we attempt to summarize seed size QTL identified in four major cucurbits (watermelon, pumpkin/squash, cucumber, and melon). From these QTL, we inferred consensus seed size (SS) QTL across different cucurbits. We found structural and functional conservation of consensus SS QTL, as well as unique QTL in each crop. Through comparative analysis across four major cucurbits, we identified potential candidate genes in syntenic regions among several major-effect SS QTL.

## Overall View of the Genes and Regulatory Networks Controlling Seed Size Variation in Plants

In angiosperms, a mature seed is composed of three parts: the embryo, the endosperm and the seed coat, all of which contribute to seed size. Seed development involves many complex processes and numerous genes (e.g., Chaudhury et al., 2001; Sun et al., 2010; Figueiredo and Kohler, 2014; Li and Li, 2016). Mutations in many of those genes may result in seed size change. Large amount of work has been done to understand the genetic control of seed size in primarily the model plants such as *Arabidopsis thaliana*, and rice (*Oryza sativa* L.). Major genes and regulatory pathways that play important roles in seed size control in plants are summarized in Supplementary Table S1. Major pathways include the ubiquitin pathway (Li et al., 2008; Xia et al., 2013; Li N. et al., 2019), the *IKU* pathway (Luo et al., 2005; Zhou et al., 2009; Wang et al., 2010), and hormone signaling pathways (Okushima et al., 2005; Du et al., 2017; Hu et al., 2018; Assefa et al., 2019), as well as other regulators (e.g., Huang et al., 2011; Wang S. et al., 2012; Wang et al., 2015; Si et al., 2016).

Several ubiquitin pathway genes have been shown in control of seed size (Li et al., 2008). The ubiquitination process involves ubiquitin activating enzymes (E1s), ubiquitin conjugating enzymes (E2s), and ubiquitin ligases (E3s) (Li et al., 2018b). In Arabidopsis, *DA1* encodes a ubiquitin receptor and acts as a negative regulator in seed size and organ development (Li et al., 2008; Dong et al., 2017). *DA2* encodes a RING-type E3 ligase with a similar function as *DA1* (Xia et al., 2013). The rice *GRAIN WIDTH AND WEIGHT2* (*GW2*) is a homolog of *DA2*; over-expression of *GW2* and *DA2* restricts seed/organ size (Song et al., 2007; Xia et al., 2013; Dong et al., 2017). The maize *ZmGW2-CHR4* and wheat *TaGW2* are homologs of rice *GW2*, both of which have also been shown to control grain size (Li et al., 2010; Su et al., 2011). In addition, the *Brassica napus* HECT E3 ligase gene *BnaUPL3.C03* regulates seed weight; its down-regulation leads to higher seed weight per pod (Downes et al., 2003; Charlotte et al., 2019).

In Arabidopsis, three genes, *HAIKU1* (*IKU1*), *HAIKU2* (*IKU2*), and *MINISEED3* (*MINI3*) in the same HAIKU (IKU) pathway coordinate to control seed size by influencing endosperm development during early seed growth (Li N. et al., 2019). *IKU1* is a VQ motif-containing protein (Wang et al., 2010). *IKU2* and *MINI3* encode a leucine-rich repeat (LRR) kinase, and a WRKY transcription factor, respectively (Garcia et al., 2003; Luo et al., 2005). The *IKU1*, *IKU2* and *MINI3* mutants all show reduced endosperm size, and thus smaller seeds (Garcia et al., 2003; Luo et al., 2005; Wang et al., 2010). In addition, *SHORT HYPOCOTYL UNDER BIUE1* (*SHB1*) is an upstream regulatory factor affecting the expression of *MINI3* and *IKU2* by interacting with other proteins in the early stage of seed development leading to enlarged seed cavity and improved endosperm growth (Zhou et al., 2009). Over-expression of *IKU2* in canola (*B. napus*) increases seed size and seed yield (Xiao et al., 2016).

Phytohormones, such as brassinosteroids (BRs), gibberellin (GA), auxin and cytokinin (CK) play important roles in seed development which directly control seed size by enhancing cell proliferation, affecting embryo and endosperm development (Jiang et al., 2013; Du et al., 2017; Hu et al., 2018; Assefa et al., 2019; Li Y. D. et al., 2019). Many genes regulate seed development through integration into plant hormone metabolism. For example, *ARF2* (*AUXIN RESPONSE FACTOR 2*) regulates seed size by limiting cell proliferation in the maternal integument (Okushima et al., 2005; Schruff et al., 2005). SRS5 (SMALL AND ROUND SEED), an α-tubulin protein, positively regulates grain length independent of the BR signaling pathway (Segami et al., 2012, 2017). In soybean, over-expression the P450 gene *GmCYP78A5* results in increasing seed weight (Du et al., 2017). The rice gene *qTGW3* encodes OsSK41 that interacts with *OsARF4* (*AUXIN RESPONSE FACTOR 4*); loss-of-function mutant of OsSK41 has increased grain length and weight (Hu et al., 2018). The grape gene *VvHB58* has a potential function in regulating seed number and development by impacting auxin, gibberellin, and ethylene signaling pathways (Li Y. D. et al., 2019). In soybean, *Glyma.02g04660* encodes a histidine phosphotransfer protein ortholog that controls seed width through the cytokinin-mediated regulating pathway (Assefa et al., 2019). In *B. nupus*, *BnaA9.CYP78A9* positively regulates seed length by affecting auxin metabolism (Shi et al., 2019).

Many transcription factors have been shown to play important roles in regulating seed development. For example, *APETALA2* (*AP2*), a member of the AP2/EREBP transcription factor gene family, regulates cell expansion in the maternal integument of seed development (Jofuku et al., 2005; Zhang et al., 2016). *AINTEGUMENTA* (*ANT*) is another type of *AP2* transcription factor affecting the seed size by regulating the cell division of integument during ovule development (Mizukami and Fischer, 2000). *TRANSPARENT TESTA GLABRA2* (*TTG2*) encodes a WRKY transcription factor that is mainly expressed in the endosperm and integument with a similar function as *AP2*: it may also affect the seed size by directly regulating integument cell elongation (Johnson et al., 2002; Garcia et al., 2005). The MADS-box genes have been found to control seed size in rice. For example, *OsMADS29* regulates development of the integument or seed coat, and *OsMADS87* may affect seed size in endosperm cellularization period (Prasad et al., 2010; Yang X. et al., 2012; Chen et al., 2016). The *WG7* (*WIDE GRAIN 7*) up-regulates *OsMADS1* expression by directly binding to its promoter, and enhancing histone H3K4me3 enrichment in the promoter and ultimately increases grain width in rice (Huang et al., 2019). The *SlPRE2* encodes a bHLH family transcription factor, which mediates plant response to gibberellin; silencing of *SlPRE2* decreases tomato seed size by restricting cell expansion (Zhu et al., 2019).

Many other genes or QTL not belonging to above-mentioned categories have been cloned that affect seed/grain size. For example, the rice gene *Os02g0192300* for a zinc finger protein seems to control grain weight (Huang et al., 2011). The rice grain width QTL *GW8* encoding OsSPL16 increases grain width by promoting cell proliferation and grain filling (Wang S. et al., 2012). *GLW7* (*GRAIN LENGTH AND WEIGHT ON CHROMOSOME 7*) also encoding a SPL protein (OsSPL13) positively regulates grain size by increasing spikelet hull cell expansion (Si et al., 2016). The rice *GW7* (*GRAIN WIDTH7*) controls grain size and shape through modulating cell proliferation (Wang et al., 2015). The rice *GS3* (*GRAIN SIZE 3*) also could regulate grain size and weight (Li et al., 2016; Zeng et al., 2020). In Arabidopsis, the cyclin gene *CYCB1;4* controls the final seed size by regulating the cell cycles in maternal tissues and zygotic tissues (Ren et al., 2019).

## QTL for Seed Size (SS) Variation in Major Cucurbit Crops

There is rich genetic diversity in seed size among major cucurbits (Figure 1), which was the result of selection during domestication or diversifying selection in breeding to adapt to crop production for various purposes (Tan et al., 2013; Wang et al., 2014; Li et al., 2018a; Zhang et al., 2018). In recent years, a number of seed size QTL mapping studies have been conducted in watermelon, pumpkin/squash, cucumber and melon QTL, which allows a comparative analysis of the genetic basis of seed size variation across different cucurbits. However, the names of the QTL are confusing in the literature. For convenience, following recommendations for QTL naming by Pan et al. (2020) and Wang et al. (2020), here we developed a QTL nomenclature for naming QTL for seed-related traits, which are presented in Table 1. In brief, SL, SWD, ST, and 100SWT stood for the seed length, seed width, seed thickness and 100-seed weight, respectively. The QTL was named according to Pan et al. (2020) which included information for its location and relative order on chromosome (e.g., *sl1.1* presents the first QTL on chromosome 1). In each crop, the same QTL could be identified in different studies with different genetic backgrounds or environments. By examining their physical locations and LOD support intervals, it is possible to establish a consensus SS QTL for these related traits (Pan et al., 2020). Thus, a consensus SS QTL will be defined based on individual SL, SWD, ST, or 100SWT QTL.

**TABLE 1:** Nomenclature of seed size QTL used in the present research.

QTL type	Traits name	Abbreviation	QTL name	Description of QTL name
Single QTL	Seed length	SL	*sl1.1*	First sl QTL on Chromosome 1
	Seed width	SWD	*swd2.2*	Second swd QTL on Chromosome 2
	Seed thickness	ST	*st3.3*	Third st QTL on Chromosome 3
	100 seed weight	100SWT	*100swt4.1*	First 100swt QTL on Chromosome 4
Consensus QTL	Watermelon seed size	ClSS	*ClSS 1.1*	First consensus SS QTL on watermelon Chromosome 1
	Cucumber seed size	CsSS	*CsSS 4.2*	Second consensus SS QTL on cucumber Chromosome 4
	Melon seed size	CmSS	*CmSS 3.1*	First consensus SS QTL on melon Chromosome 3

### Seed Size QTL in Watermelon

Watermelon could be divided into two types based on its uses: use of the edible flesh and use of seeds. For seed-use watermelons, large seeds are preferred because they are rich in oils and proteins, and easy to crack open (Al-Khalifa, 1996; Baboli and Kordi, 2010; Prothro et al., 2012b), while for edible flesh watermelons, small and few (or no) seeds could increase the pulp proportion and improve product quality. Thus, seed size is a target of selection in watermelon breeding, but the breeding direction of selection depends on its end use.

There is a wide range of variation in seed size among watermelon collections (e.g., Figure 1A). The watermelon seed could be classified into six representative types based on the size: giant, big, medium, small, tiny, and tomato seed (Yongjae et al., 2009). Poole et al. (1941) were pioneers who paid attention to the inheritance of seed size. They found that the length of large and medium-sized seeds was controlled by two genes (*l* and *s*), and that small seeds were dominant to medium seed size. Later, two gene, *Ti* for *tiny seed* and *ts* for *tomato seed* were also reported (Zhang et al., 1994; Tanaka et al., 1995; Zhang, 1996). Zhang and Zhang (2011) found that a pair of major genes and a pair of recessive genes determine seed size, but additional modifiers are possible. Kim et al. (2015) considered watermelon seed size as a quality trait that was controlled by a single dominant gene. Gao et al. (2016) carried out genetic analysis on seed size-related traits, and found that seed length, width and 1000-seed weight were quantitatively inherited that show continuous variation in segregating populations, and they may be controlled by major QTL. The quantitative nature of seed size variation in watermelon was further observed in other studies (e.g., Prothro et al., 2012a; Zhou et al., 2016). Prothro et al. (2012a) were probably the first to conduct QTL analysis for seed size in watermelon, but pre-draft genome studies were sporadic (Meru and McGregor, 2013). Using a recombinant inbred line (RIL) population from KBS (medium seed, cultivated) × NHM (medium seed, cultivated) and an F_2_ population from ZWRM (small seed, cultivated) × PI244019 (medium seed, *Citroides*), Prothro et al. (2012a) conducted QTL mapping on seed size related traits and identified 13 QTL on four linkage groups (LG2, LG4, LG9 and LG11). In both populations, three major QTL with phenotypic variance explained (PVE) being from 26.9 to 73.6% for SL, SWD and 100SWT, were identified at the same region on Chr6 (LG2). In addition, they also detected a major-effect QTL (PVE = 25.6%) for SWD on Chr2 (LG9). Using an F_2_ population from the normal type (PI 279261) × egusi type (PI 560023) cross, Meru and McGregor (2013) detected 3 major-effect QTL (PVE = 34.4–60.8%) on Chr6, which seem to be the same chromosomal region as identified by Prothro et al. (2012a).

The release of the watermelon draft genome (Guo et al., 2013) greatly facilitated QTL mapping studies. Ren et al. (2014) verified the major QTL on Chr2 and Chr6 reported by Prothro et al. (2012a). Several studies all detected SS-related QTL in the same Chr6 region (Sandlin et al., 2012; Prothro et al., 2012a, b; Ren et al., 2012). Meanwhile, using an F_2_ population developed from the cross between wild egusi type (PI 186490) and cultivated type (LSW-177) watermelons, Liu et al. (2014) identified 1 SL QTL (*QSL*) and 1 SWD QTL (*QSWD1*) on Chr6 (PVE = 18.8 and 15.7%), respectively. With the same population, Zhou et al. (2016) mapped more SS QTL (PVE = 3.2–28.9%) on Chr6 including 3 SL, 4 SWD, 1 ST, and 2 100SWT QTL. Kim et al. (2015) detected a 20-seed-weight QTL on Chr2 with an F_2_ population from the cross between the open-pollinated cultivar ‘Arka Manik’ and Jubilee-type inbred line ‘TS34.’ In our recent study, we identified a major-effect QTL, *SS6.1* for seed size on Chr6 using an F_2_ population developed from the cross between a small size line and a medium size line. The PVE for QTL of SL, SWD, and 100SWT in this population was as high as 48.5, 42.2, and 45.3%, respectively. Interestingly, in an F_2_ population from the cross between two watermelon inbred lines with medium size seeds, we detected a major-effect QTL (PVE = 35.5–50.1%) for SL, SWD, and 20SWT (20 seed weight) on Chr2 (Gao et al. unpublished data).

Li et al. (2018a) conducted fine mapping of QTL for seed-related traits in watermelon, and identified a major-effect QTL *qSS6* for seed size. The PVE of the QTL for SL, SWD, and 1000SWT (1000-seed weight) in this population were as high as 94.1, 95.3, and 93.0%, respectively. They further identified a region of *qSS6* harboring three candidate genes including *Cla009291*, *Cla009301*, and *Cla009310*. Among them, *Cla009291* encodes the MDR protein mdtK, which is differentially expressed at different seed developmental stages between large- and small-seeded lines. *Cla009301* is a homolog of *SRS3* (*SMALL AND ROUND SEED*) for a BY-kinesin-like protein 10 that is a seed size regulator in rice (Kanako et al., 2010). *Cla009310* encodes an unknown protein that was proposed to be a candidate for *qSS6* based on an SNP in the first exon between the two parental lines (Li et al., 2018a).

**FIGURE 2:**
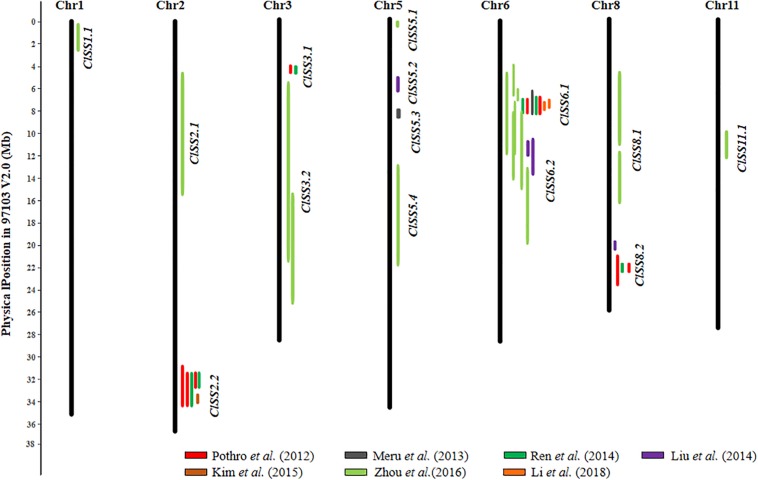
Distribution of 14 seed size (SS) consensus QTL in the watermelon (97103 V2.0) draft genome. Physical LOD intervals of QTL identified from different studies are presented to the right of each chromosome. QTL detected by the same study are showed with same color. Only chromosomes harboring seed size QTL are shown.

To summarize, so far 20 SL, 19 SWD, 3 ST, and 19 100SWT QTL (total 61) have been identified in watermelon. The details of these QTL are presented in Supplementary Table S2. Additional information for each QTL is also provided including flanking markers with sequences, their chromosomal positions, mapping populations and PVE are presented in Supplementary Table S2. Based on their chromosomal locations and LOD support intervals, 14 consensus SS QTL were inferred from different studies. The information of all 14 consensus SS QTL including independent QTL from each study is listed in Supplementary Table S3, their chromosomal positions are visually illustrated in Figure 2. The 14 consensus SS QTL were distributed on 7 of the 11 watermelon chromosomes. Of them, 4 QTL (*ClSS2.2*, *ClSS6.1*, *ClSS6.2*, and *ClSS8.2*) could be detected in at least two populations/studies. *ClSS2.2* was only detected by the populations constructed from two cultivated lines, but *ClSS6.1* and *ClSS6.2* could be detected in populations derived from cultivated and wild watermelon lines. Especially, *ClSS6.1* and *ClSS6.2* were identified in most studies with F_2_ and RILs populations derived from different seed size materials, suggesting that this QTL may play the most important role in seed size/weight determination. Meanwhile, some ‘consensus’ QTL such as *ClSS1.1* and *ClSS11.1* were only detected in a single population/study (e.g., Zhou et al., 2016), which should be considered putative pending validation in future studies.

### Seed Size QTL in Pumpkin/Squash

Pumpkin/squash (*Cucurbit* spp.) are important cucurbit crops cultivated worldwide. The seed size of seed-use pumpkin affects not only seed yield, but may also impact fruit quality. For example, Paris and Nerson (2003) found that pumpkin SL and SWD were positively correlated with the fruit size, while the seed shape was negatively correlated with the fruit shape. There was larger heterosis of SWD than that of SL (Xu et al., 2006). Seed number per fruit and 100SWT directly affect the yield of seed-use pumpkin (Wang P. et al., 2012). Meru et al. (2018) found that seed size was positively correlated with oil content, but negatively with seed protein in pumpkin. However, little is known about the genetic basis of seed size variation in pumpkin/squash. So far, relatively few QTL have been identified underlying seed size variation in pumpkin/squash. Tan et al. (2013) developed an F_2_ population using the Indian large-grain ‘0515-1’ and the small-grain ‘0460-1-1’ pumpkin lines; QTL analysis detected 4 major-effect QTL controlling SWD on LG2, LG3 and LG4 with PVE from 2.9 to 29.7%. In *C. maxima*, Wang et al. (2019) developed a high-density genetic map with the F_2_ population from the cross between two squash inbred lines (‘2013-12’ and ‘9-6’). In QTL mapping, they identified 10 QTL on six chromosomes (Chr) (Chr4, 5, 6, 8, 17, and 18), including 4 for SL, 4 for SW, and 2 for HSW (100-hundred-seed weight) with the PVE ranging from 7.0 to 38.6%. The major-effect QTL *SL6-1* was located on Chr6, and explained 38.6% of observed phenotypic variance. Another major-effect QTL, *SW6-1* was also detected on Chr6 with the PVE of 28.9%. The details of these QTL are presented in Supplementary Table S4. Since the limited number of seed size-related QTL were detected, no consensus QTL could be inferred in pumpkin/squash.

### Seed Size QTL in Cucumber

A few studies have been conducted in cucumber for QTL mapping of seed size-related traits. Chen et al. (2012) suggested that SL in cucumber is a quantitative trait controlled by multiple genes. With a RIL population developed from the cross between two cultivated cucumber lines: the large seeded ‘D06157’ and small seeded ‘D0603,’ they detected 6 SL QTL. In the RIL population derived from PI 183967 (wild) × 931 (cultivated), Wang et al. (2014) detected 14 SS QTL on five chromosomes (Chr2, 3, 4, 5, and 6) with mainly additive effect including 6 for SL (PVE = 7.5–15.6%), 4 for SWD (PVE = 7.7–18.8%), and 4 for 100SWT (PVE = 11.0–28.3%). However, only *SL5.1*, *SL6.1*, and *100SWT6.1* could be detected in two seasons. In another study, using an F_2__:__3_ population derived from the cross between two US processing cucumber inbred lines ‘2A’ and ‘Gy8,’ Lietzow (2015) identified 8 QTL for SS and 50SWT (50 seed weight) with two major QTL (PVE = 11.9% for both) located on Chr4.

**FIGURE 3:**
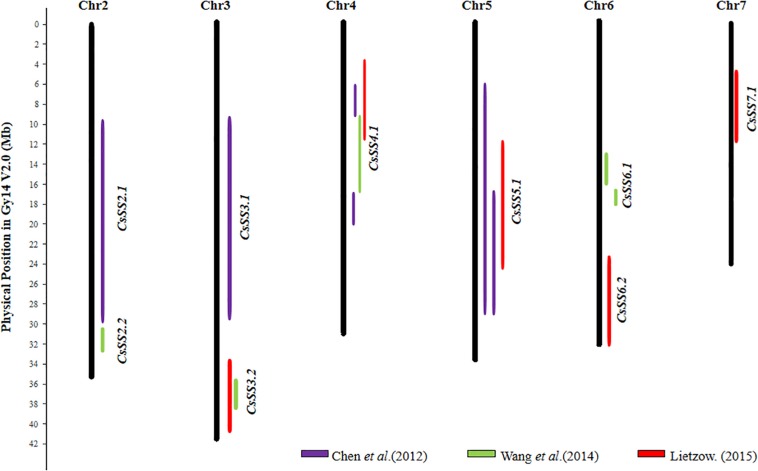
Distribution of 9 seed size (SS) consensus QTL in the cucumber (Gy14 V2.0) draft genome. Physical LOD intervals of QTL identified from different studies are presented to the right of each chromosome. QTL detected by the same study are showed with same color. Only chromosomes harboring seed size QTL are shown.

From the 28 QTL identified in cucumber (details in Supplementary Table S5), 9 consensus SS QTL could be established, which are listed in Supplementary Table S6. Their chromosomal locations are graphically presented in Figure 3. Five of the 9 consensus SS QTL, *CsSS2.2*, *CsSS3.2*, *CsSS4.1*, *CsSS5.1*, and *CsSS6.1* were identified with populations derived from crosses between the wild and cultivated cucumbers, of which *CsSS4.1* and *CsSS5.1* were detected in all studies with segregating populations created by parental materials with different seed sizes and origins. Therefore, *CsSS4.1* and *CsSS5.1* may be the potential target regions for selection during both domestication and diversifying selection.

### Seed Size QTL in Melon

Five studies conducted QTL mapping for seed size in melon. Jiao (2017) conducted QTL analysis for seed size with an F_2__:__3_ population derived from the cross of large seeded ‘ms-5’ and small seeded ‘HM-1.’ 7 QTL for SL, SWD, and 100SWT were identified on four chromosomes (Chr5, 6, 9, and 11). The 2 major-effect QTL, *Sl11.1* and *SW11.1* for SL and SWD were detected in the same region on Chr11 with PVE of 17.5 and 19.5%, respectively. With a BC_1_ population from ‘MR-1’ (long seed) × ‘M1-15’ (short/narrow seed), Ye et al. (2017) detected 4 QTL, including 2 for SL, 1 for SWD and 1 for 100SWT. The 2 QTL (*SL5.1* and *SD5.1*) on Chr5 (PVE = 9.9–11.2%), and 2 QTL (*SL1.1* and *TGW1.2*) on Chr1 were validated. In addition, using a BC_1_ population, Ning et al. (2018) also detected QTL for SL and 100SWT on Chr1 (LG1), but not in the same region reported by Ye et al. (2017). With a high-density genetic linkage map constructed by a RIL population, Pereira et al. (2018) identified 4 QTL for SWD with 1 major QTL (*SWQU8.1*) located on Chr8 with PVE of 19.9%. Zhang et al. (2018) mapped 6 QTL on SL, SWD and 100SWT in a F_2__:__3_ population from the cross between cantaloupe line ‘ms-5’ with large seed and muskmelon line ‘HM1-1’ with small seed, the major QTL (*sl11.1* and *sd2.1*) for SL and SWD were identified on Chr11 and Chr2 (PVE = 17.5 and 18.1%), respectively. Three QTL (*sl11.1*, *sl11.2*, and *100swt11.1*) for SL and 100SWT were detected in the same Chr11 region.

**FIGURE 4:**
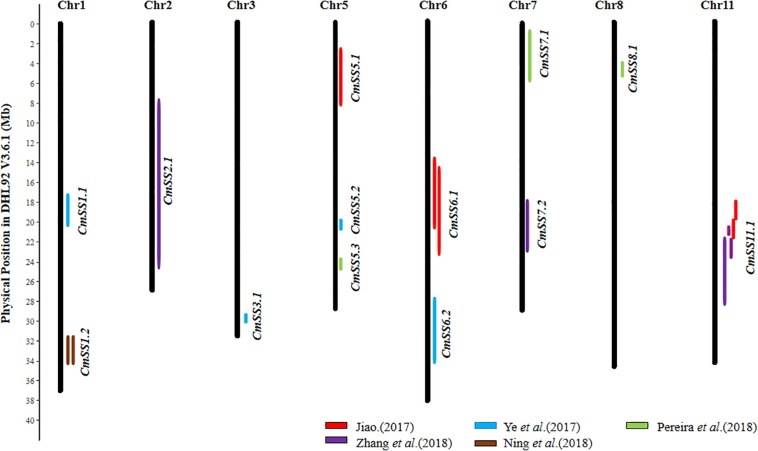
Distribution of 13 seed size (SS) consensus QTL in the melon (DHL92 V3.6.1) draft genome. Physical LOD intervals of QTL identified from different studies are presented to the right of each chromosome. QTL detected by the same study are showed with same color. Only chromosomes harboring seed size QTL are shown.

From 23 QTL detected in the six studies (Supplementary Table S7), 13 consensus SS QTL could be inferred, and the details are provided in Supplementary Table S8, and their chromosomal locations are illustrated in Figure 4. Both Jiao (2017) and Zhang et al. (2018) detected the QTL for SL, SWD, and 100SWT in the same region on Chr11, and other QTL were not detected reproducibly. Therefore, we speculated that the *Cm11.1* may play a major role in regulating seed size/weight variation in melon.

## Structure and Function Conservation of SS QTL in Major Cucurbits

**FIGURE 5:**
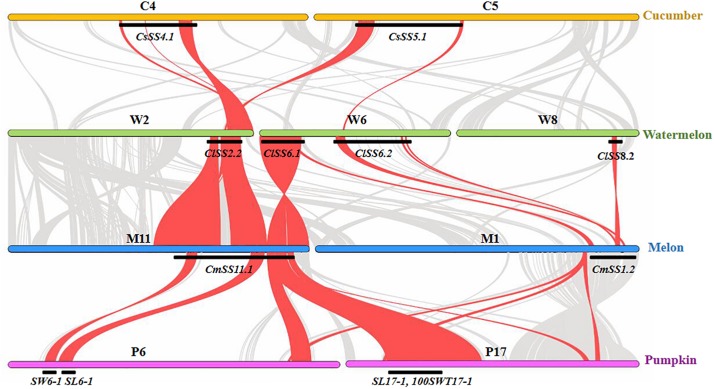
Syntenic relationships of watermelon chromosomes W2, W6, and W8 harboring major-effect consensus SS QTL with cucumber, melon and pumpkin chromosomes. The inference is based on watermelon “97103 V2.0,” cucumber “Gy14 V2.0,” melon “DHL92 V3.6.1,” and pumpkin “Rimu” draft genomes.

Previous studies have revealed high degree of sequence conservation and synteny across different cucurbit genomes, and the syntenic relationships among different chromosomal regions have also been well-established (e.g., Li et al., 2011; Garcia-Mas et al., 2012; Guo et al., 2013; Yang et al., 2014; Zhu et al., 2016; Pan et al., 2020). Comparison of consensus SS QTL locations identified from the present study may help reveal possible conservation of the genetic basis of seed size variation across major cucurbit crops. In total, 14, 9, and 13 consensus SS QTL were established based on their physical positions in the watermelon, cucumber, and melon draft genomes, respectively (Figures 2–4). Among the four major cucurbits, watermelon has the most extensive studies on QTL mapping of seed size related traits (Figure 2 and Supplementary Tables S2, S3). Four watermelon SS QTL have been repeatedly detected in multiple populations and environments, including *ClSS2.2*, *ClSS6.1*, *ClSS6.2*, and *ClSS8.2* (Figure 2). To further investigate the syntenic relationships of these major-effect, highly stable SS consensus QTL with those in other cucurbits, we aligned watermelon chromosomes 2 (W2), 6 (W6) and 8 (W8) with cucumber, melon and pumpkin genomes. We looked into the syntenic relationships of the three watermelon chromosomes (W2, W6, and W8) in cucumber, melon and pumpkin/squash. We searched the syntenic blocks based on the physical positions of these consensus QTL in the cucurbits draft genomes, which could be conveniently conducted through the cucurbit genome database at http://cucurbitgenomics.org/. The syntenic relationships of the three watermelon chromosomes (W2, W6, and W8) in cucumber, melon and pumpkin/squash were calculated by the One Step MCScanx function, and the syntenic blocks were drawn by the visualization tools Dual Systeny Pltter function in TBtools software. The syntenic relationships of the three watermelon chromosomes with those in other three cucurbits are illustrated in Figure 5. The syntenic blocks harboring the four watermelon SS QTL and those in melon, cucumber, and pumpkin are highlighted in red. From Figure 5, it is easy to see that the chromosomal region harboring watermelon *ClSS2.2* (W2) was syntenic to the regions where the cucumber *CsSS4.1* (C4) and *CsSS5.1* (C5), melon *CmSS11.1* (M11) and pumpkin *SL6-1*, *SW6-1* (P6), were respectively located. *CmSS11.1* (M11) and *CsSS4.1* (C4) were also in syntenic regions. Similarly, *ClSS6.1* (W6) was syntenic to the regions *CmSS11.1* (M11) in melon and pumpkin *SL17-1*, *100SWT17-1* (P17). *CmSS1.2* (M1) and the regions harboring both *ClSS6.1* (W6) and *ClSS8.2* (W8) were syntenic. These observations suggested possible conservation of functions and structure genes/QTL for seed size control among cucurbit crops. Such conservation could also be possible for QTL in other regions. However, more QTL mapping work is needed to make such inferences.

The molecular mechanisms of seed size regulation in cucurbits are unknown. The functions of many genes for organ development are high conserved (for example, fruit size and shape as reviewed in Pan et al., 2020). From QTL mapping studies, the four watermelon consensus SS QTL (*ClSS2.2*, *ClSS6.1*, *ClSS6.2*, and *ClSS8.2*) have been delimited to relatively small regions. We examined the annotated genes in the four regions from the watermelon draft genomes. In the watermelon 97103 V2.0 draft genome, 21, 11, 6, and 34 genes were predicted in the *ClSS2.2*, *ClSS6.1*, *ClSS6.2* and *ClSS8.2* consensus QTL regions, respectively, which are listed in Supplementary Table S9. Among those genes, several seem to be good candidates for these SS QTL. As discussed early (see section “Introduction,” Supplementary Table S1), the ubiquitination pathway may play important roles in control of seed size (e.g., Song et al., 2007; Li et al., 2010; Su et al., 2011; Xia et al., 2013; Wang et al., 2016; Dong et al., 2017). From our recent study in pumpkin, the E3 ubiquitin-protein ligase gene was located in the *SL6-1* and *SW6-1* major-effect QTL region (Wang et al., 2019). In rice, a gene encoding a zinc finger protein has been verified in control grain weight (Huang et al., 2011). In the watermelon SS QTL regions, there were six genes with predicted functions to encode zinc finger proteins including *Cla97C02G045430*, *Cla97C02G045460* and *Cla97C02G045500* in the *ClSS2.2* region, *Cla97C06G114460* in *ClSS6.1* region, and *Cla97C08G154340* and *Cla97C08G154360* in *ClSS8.2* region. Another gene, *Cla97C08G154570* encoding E3 ubiquitin-protein ligase was located in *ClSS8.2* region (Supplementary Table S9). These genes might be interesting candidates for the seed size QTL that merit consideration in future studies.

## Conclusion Remarks

In this study, we summarized QTL for seed size-related traits in major cucurbits, and identified consensus SS QTL in watermelon, melon and cucumber. Many of these SS QTL were located in non-syntenic regions in the three cucurbit crops. In watermelon and melon, it seems that several stable consensus QTL are located in syntenic blocks in different cucurbit crops (Figure 5), which may suggest shared common QTL for seed size control in some genetic backgrounds. It should be noted that QTL mapping studies for seed size/weight are very limited in major cucurbit crops (none in minor cucurbits). Many of these consensus QTL were inferred from single study. The genomic regions harboring these QTL are still very large. The effects of these QTL need to be confirmed, and their locations need to be refined in future studies. Seed size/weight was the target of selection during domestication from wild ancestors, which usually have small seeds. In seed-use cucurbits like watermelon and pumpkin, seed size may be under further selection during breeding, while in melon and cucumber where flesh (endocarp) is consumed, seed size may not be a main target in breeding. Understanding the genetic basis and the roles of selection in diversifying and domestication shaping seed size variation might be some interesting topics in future studies. Such information is also important in marker-assisted selection in breeding for seed size in cucurbit crops.

## Author Contributions

YGu and MG conducted literature review and wrote the manuscript. XXL, MX, and XSL performed the comparative analysis. FL and SQ helped review of watermelon, melon, and pumpkin data. YZ, JL, XJL, and YGa analyzed and reviewed cucumber and melon data. All authors read and approved the final submission.

## Conflict of Interest

The authors declare that the research was conducted in the absence of any commercial or financial relationships that could be construed as a potential conflict of interest.
